# Postmarketing all-case surveillance trends and contribution to safety measures of drugs approved in Japan: a cross-sectional survey in 1999–2019

**DOI:** 10.1007/s11096-022-01461-0

**Published:** 2022-11-02

**Authors:** Minami Nakao, Yuri Nakamura, Masafumi Shimokawa, Hideki Maeda

**Affiliations:** 1 Department of Regulatory Science, Faculty of Pharmacy, Meiji Pharmaceutical University, 2-522-1, Noshio, Kiyose-City, Tokyo 204-5255 Japan; 2 Faculty of Pharmaceutical Sciences, Sanyo-Onoda City University, Yamaguchi, Japan

**Keywords:** Japan, Postmarketing all-case surveillance, Regulatory science, Risk management plan, Safety

## Abstract

**Background:**

Postmarketing all-case surveillance (PACS) is a safety monitoring activity predominantly conducted for drugs with few domestic clinical trials, orphan drugs, or anticancer drugs that potentially cause serious adverse events.

**Aim:**

This study comprehensively analyzed drugs in Japan requiring PACS as an approval condition and those implementing PACS-results-based safety measures.

**Method:**

We included drugs approved in Japan between 1999 and 2019.

**Results:**

During the 20-year survey, 1871 drugs were approved in Japan, including 277 (14.8%) requiring PACS as an approval prerequisite. The drug number requiring PACS for approval and its ratio to the total approved-drug number is increasing annually. In 2018, the number and percentage of PACS-requiring drugs reached a 37-drug maximum (32.5%). Additionally, among the 277 PACS-requiring drugs, upon examining the results of 87 drugs for which reexamination results had already been obtained, all 87 drugs (31.4%) were found to be in Category 1 which means there is no need to revise drug-approval conditions, indicating that their usefulness is consistent with approval. Furthermore, measures such as revising the package insert and providing information to medical institutions were adopted for 53 drugs, 14 of which had PACS-results-based safety measures.

**Conclusion:**

PACS implementation for drug approval will potentially continue increasing. Normally, PACS is not conducted overseas, as it is a safety-monitoring activity exclusive to Japan, and the burden on institutions, such as medical sites and pharmaceutical companies, is heavy. Thus, ensuring a balance between the obtained effect and this burden is imperative.

**Supplementary Information:**

The online version contains supplementary material available at 10.1007/s11096-022-01461-0.

## Impact statements

- In this study, the characteristics of postmarketing all-case surveillance drugs in Japan for the past 20 years, since the introduction of postmarketing all-case surveillance, were comprehensively surveyed and analyzed.
- The results of this study allow us to understand what kind of drugs have implemented postmarketing all-case surveillance.
- The findings also facilitate our understanding of how the results of postmarketing all-case surveillance were utilized in safety measures.

## Introduction

In recent years, while innovative drugs have been developed for diseases with excessive medical demands, when attempting to obtain strong evidence in a clinical trial, patient recruitment and conducting the actual trial are potentially time consuming. This may result in longer development periods and delayed access to care for patients. For diseases with particularly high medical demands, the effect of prolonged development on patient access is significant, and policies that expedite patient access to drugs as much as possible, while continuing to ensure their efficacy and safety, are warranted. In each country, systems, such as early approval systems [1–4] and conditional early approval systems [5–7], have been established, and drugs are often approved without conducting confirmatory clinical trials (waiver of confirmatory clinical trials), thus decreasing the amount of data available before a drug’s approval [8]. Consequently, approval is granted on condition that clinical trials are conducted, and safety measures are adopted after drugs become commercially available.

In 1995, irinotecan was the first to require post-marketing all-case surveillance (PACS) as a condition for approval [9]. Thereafter, PACS became widespread from around 1999 as a post-marketing safety measure [10]. PACS is a system of safety monitoring activities unique to Japan that are not found in the United States or Europe. PACS is required for the approval of orphan drugs when the number of clinical trials conducted in Japan is negligible or absent as well as for antineoplastic drugs that potentially cause serious adverse events where the number of clinical trials conducted is insignificant [11, 12]. It is necessary to collect information on all patients who received drugs during a certain period when PMDA and pharmaceutical companies agree or until a target number of surveillance is reached, and safety-related data are collected by medical representatives (MRs) who are sales persons of pharmaceutical company [12]. One advantage of investigating all cases is that it is possible to collect safety information after a drug becomes commercially available without bias at an early stage. However, disadvantages, such as the increased burden on medical professionals in having to provide manpower to assist in research activities and increased cost incurred by pharmaceutical companies, have been highlighted. Moreover, Japan’s unique safety monitoring activities are a heavy burden, especially for foreign-affiliated companies in their global drug development.

Additionally, although PACS is not well known overseas, approximately 20 years have elapsed since its introduction. Presumably, several drugs have thus far required PACS as a condition for their approval and have been approved on such grounds in Japan. However, the details of its progress have rarely been reported. To our knowledge, there are no reports of a long-term and comprehensive studies about the situation of PACS other than this study, and reports are written exclusively in Japanese [13]. Many uncertainties exist regarding recent trends of drugs requiring PACS as a condition for their approval as well as how PACS results are reflected in safety measures. Moreover, since PACS is unique to Japan, there has been almost no dissemination to other countries.

### Aim

This study comprehensively analyzed drugs in Japan requiring PACS as an approval condition and those implementing PACS-results-based safety measures.

### Ethics approval

This study did not require institutional review board approval or patient informed consent because it was based on publicly available information involving no patient records.

## Method

### Data construction

In this study, prescription drugs approved in Japan between September 1999 and December 2019 were surveyed. Initial new drug applications (iNDAs) as new molecular entities and supplemental NDAs (sNDAs) for additional indications were included in the survey. This study was prepared according to the Strengthening the Reporting of Observational Studies in Epidemiology reporting guidelines [14] for cross-sectional studies.

### Data collection and regulatory characteristics

Data were collected from publicly available databases on the Pharmaceuticals and Medical Devices Agency (PMDA) website (http://www.pmda.go.jp/english/index.html). When surveying, prescription drugs approved in Japan between September 1999 and December 2019 were initially specified. We subsequently identified drugs with conditional approval for PACS. Finally, survey items regarding the following drug background and regulatory characteristics of each drug were investigated, and an independent database was created: information on application, application type (new/additional indication/additional dosage, etc.), disease classification, therapeutic indication classification, regulatory review field (PMDA review department), review time, indication, special notes for review (expedited review/priority review/pre-review, etc.), applicant (Japanese company/foreign company), application data package, type of clinical trial data, and the presence or absence of multiregional clinical trials and information on approval were obtained and analyzed. For pediatric drugs, package inserts [15] were analyzed, and those with descriptions such as “children” and “newborn,” in the column of “dosage and administration” or “indication,” were surveyed, and drugs for orphan diseases—drugs that had undergone an orphan-disease application [16]—were also surveyed. In terms of the regulatory review field, classification was performed according to the PMDA regulatory review fields [17]; in terms of therapeutic indication, classification was performed according to the Japan Standard Commodity Classification numbers [18]; and disease classification was performed according to the International Statistical Classification of Diseases and Related Health Problems classification [19].

### Reexamination reports and how PACS results were reflected in safety measures

The reexamination reports used were those posted on the PMDA website on September 24, 2021. Drugs for which PACS was a condition for their approval were identified based on review reports, and various data, including drug-related information, background information, and regulatory information, were investigated. The following items from the reexamination reports on these drugs were also investigated and consolidated into an original database: changes in the contents of the package insert (warnings, contraindications, adverse events, etc.), provision of information to medical institutions (training), and other related information.

### Statistical analysis

Descriptive statistics were used to characterize the new drugs and their indications. We used the chi-square test to analyse the differences between in categorical variables of regulatory characteristics between PACS drugs and other drugs to determine trend comparisons for PACS drugs. All statistical tests were two-tailed, and statistical significance was set at *P* < 0.05. All analyses were performed using Microsoft Excel 2019 analytical tools.

## Results

### Investigated drugs

**Fig. 1:**
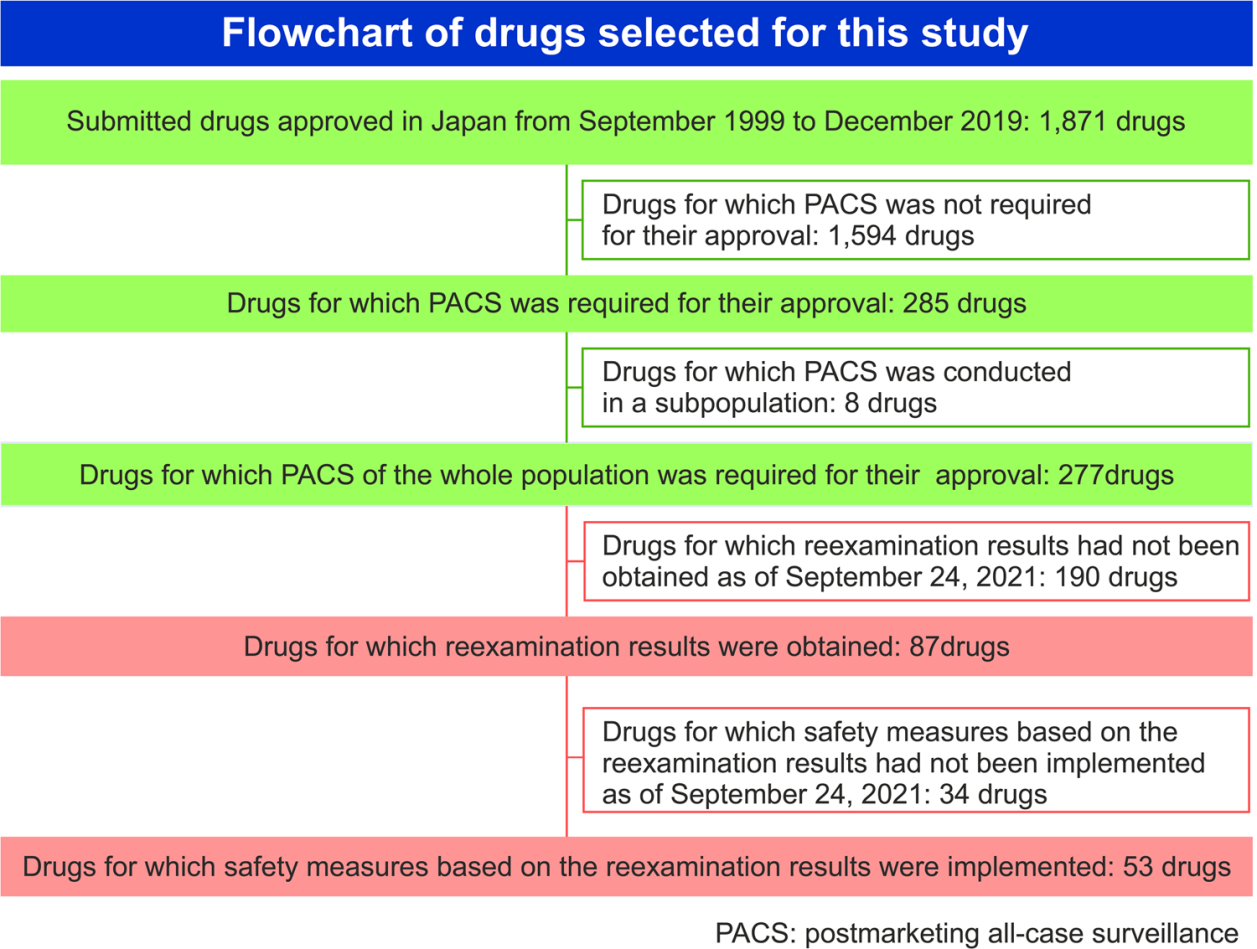
Flowchart of drugs selected for this study

During the 20-year period from September 1999 to December 2019, 1,871 prescription drugs were approved in Japan, of which 277 (14.8%) required PACS as a condition for their approval (Fig. 1). Of the drugs examined using PACS, 133 (19.7%) were submitted as new drugs (new molecular entities, iNDAs), and PACS was a requirement for their approval. Compared to the drugs submitted as sNDAs that also required PACS for their approval (144 drugs, 12.0%), there were significantly more new drugs (*p* < 0.001). (Table 1).

**Table 1:** Number of conditional approvals for postmarketing all-case surveillance and normal applications between 1999 and 2019 in Japan

	Post-marketing all-case surveillance	Normal applications	Total	Chi-square test (*p* value)
Number of approvals	277 (14.8%)	1594 (85.2%)	1871 (100.0%)	–
Initial NDAs (new molecular entities)	133 (19.7%)	542 (80.3%)	675 (100.0%)	*p* < 0.001
Supplemental NDAs	144 (12.0%)	1052 (88.0%)	1196 (100.0%)	

*NDA* New drug application

### Changes over time in drugs examined using PACS

**Fig. 2:**
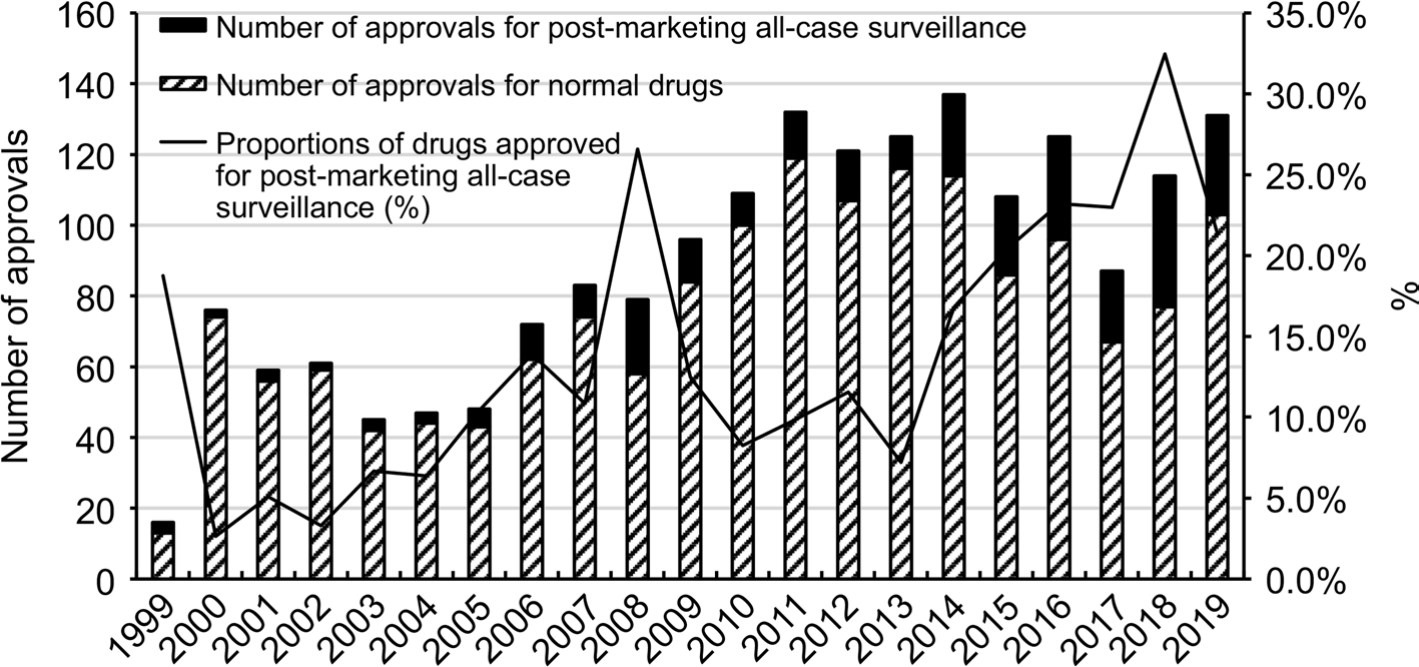
Changes in conditional approvals for postmarketing all-case surveillance and normal applications between 1999 and 2019 in Japan

On analyzing the changes over time in the number of approved drugs for which PACS was a condition for their approval, the number has been increasing annually since the approval of the first three drugs in 1999 (Fig. 2). On examining the percentage of all approved drugs, in 2008 as well as in the preceding five years, over 20% of the approved drugs were those subjected to PACS.

### Differences between the background of drugs for which PACS was a condition for their approval and that of other regular drugs

The backgrounds and regulatory characteristics of 277 drugs for which PACS was performed and 1594 drugs for which a regular application was used were investigated and compared. The results are shown in Table 2. Regarding drug-related background information and regulatory characteristics, the following were examined: regulatory review field, therapeutic indication classification, application data package, types of clinical trial data, presence or absence of international joint-research studies, applicants (Japanese/Foreign companies), pediatric drugs, orphan drugs, public applications, and preferential treatment for approval review. The results revealed that there were significantly more PACS-related-drug approvals in regulatory review fields related to anticancer (PACS vs. normal approvals; 40.1 vs 13.8%, *p* < 0.0001) and AIDS drugs (PACS vs. normal approvals; 4.3 vs. 1.5%,  *p* 0.002). However, there were significantly less PACS-related-drug approvals in the regulatory review fields related to drugs for the central nervous system(PACS vs. normal approvals; 8.3 vs. 12.5%, *p* = 0.044), infectious diseases (PACS vs. normal approvals; 3.2 vs. 12.5%, *p* < 0.0001), and the urinary system(PACS vs. normal approvals; 1.1 vs. 5.0%, *p* 0.004). Regarding therapeutic indication classification, PACS was significantly more common in anticancer drugs (PACS vs. normal approvals; 41.2 vs. 16.8%, *p* < 0.0001) and significantly less common in drugs related to the central nervous (PACS vs. normal approvals; 6.9 vs. 14.4.%, *p* = 0.001) and circulatory systems (PACS vs. normal approvals; 14.4 vs. 24.3%, *p* < 0.0001). In addition, regarding application data packages (PACS vs. normal approvals; 68.2 vs. 35.6%, *p* < 0.0001), drugs that underwent overseas and multiregional clinical trials (PACS vs. normal approvals; 32.1 vs. 10.5%, *p* < 00001) often required PACS as a condition for their approval. Moreover, PACS was significantly less common in drugs with clinical trial results obtained solely from Japan (PACS vs. normal approvals; 11.2 vs. 18.7%, *p* = 0.002) and those without clinical trials (PACS vs. normal approvals; 4.0 vs. 11.7%, *p* < 0.0001). Additionally, PACS is often a condition for approval for the following drugs: those manufactured by foreign-affiliated companies (PACS vs. normal approvals; Japanese companies: foreign companies: combination = 36.1%: 62.8%: 1.1%, vs. 47.9%: 47.1%: 5.0%, *p* < 0.0001); orphan drugs (PACS vs. normal approvals; 58.5 vs. 11.1%, *p* < 0.0001); those requiring preferential treatment for reviews, such as priority reviews (PACS vs. normal approvals; 11.2 vs. 5.1%, *p* < 0.0001); and those that received a Sakigake (pioneering) designation (PACS vs. normal approvals; 1.1 vs. 0.1%, *p* = 0.004)/conditional approval (PACS vs. normal approvals; 0.7 vs. 0.0%, *p* = 0.001). The summary of the factors associated with PACS implementation considering from these results in Table 3.

**Table 2:** Therapeutic indication and regulatory characteristics of postmarketing all-case surveillance drugs and normal drugs

		Postmarketing all-case surveillance (n = 277)		Normal approvals (n = 1,594)		*P* value
		n	%	n	%	
Regulatory Review Field	Oncology	111	40.1	220	13.8	< 0.0001
	Field 1 (Gastroenterology)	31	11.2	201	12.6	0.5085
	Field 2 (Cardiovascular Disease)	29	10.5	215	13.5	0.1684
	Field 3 (Central Nerve System)	23	8.3	200	12.5	0.0442
	Field 4 (Infectious Disease)	9	3.2	200	12.5	< 0.0001
	Field 5 (Urology)	3	1.1	79	5.0	0.0036
	Field 6 (Respiratory Diseases, Metabolic Disease)	44	15.9	309	19.4	0.1692
	Field of AIDS (Acquired Immune Deficiency Syndrome)	12	4.3	24	1.5	0.0015
	Field of blood derivatives	9	3.2	38	2.4	0.3957
	Field of diagnostic drugs	3	1.1	29	1.8	0.3830
	Field of Radiopharmaceuticals	2	0.7	10	0.6	0.8554
	Others	1	0.4	69	4.3	0.0013
Therapeutic Indication Classification	Central nerve system disease	19	6.9	229	14.4	0.0006
	Cardiovascular Disease, Hormone, Gastroenterology	40	14.4	388	24.3	0.0002
	Blood Derivatives, Metabolic Disease	53	19.1	276	17.3	0.4630
	Oncology, Radiopharmaceuticals	114	41.2	268	16.8	< 0.0001
	Infectious disease	47	17.0	350	22.0	0.0608
	Topical use drugs	3	1.1	46	2.9	0.0828
	Narcotic drug	1	0.4	27	1.7	0.0917
	Others	0	0.0	10	0.6	0.1862
Data Package	Only domestic clinical data	31	11.2	298	18.7	0.0024
	Including foreign clinical data	189	68.2	567	35.6	< 0.0001
	Bridging strategy	4	1.4	44	2.8	0.2009
	Including multi-regional clinical trials	89	32.1	167	10.5	< 0.0001
	No clinical trials	11	4.0	187	11.7	0.0001
Type of Applicant	Japanese companies	100	36.1	764	47.9	< 0.0001
	Foreign companies	174	62.8	751	47.1	
	Combination	3	1.1	79	5.0	
Others	Pediatric drugs	36	13.0	239	15.0	0.3862
	Orphan drugs	162	58.5	177	11.1	< 0.0001
	Public knowledge-based application	5	1.8	214	13.4	< 0.0001
	Expedited review/Priority Review	40	14.4	262	16.4	< 0.0001
	Sakigake designation/conditional approval	5	1.8	2	0.1	< 0.0001

**Table 3:** Summaries of the factors associated with postmarketing all-case surveillance implementation

Factors tracked by many PACS surveys (Potential Factors)	Factors tracked by few PACS surveys
“New active ingredients”	“New dosage forms”
“Antineoplastic/Anticancer drugs field” “AIDS field”	“Third Field (Drugs for the Central Nervous System and Sensory Organs )” “Fourth Field (Infectious Disease Drugs)” “Fifth Field (drugs for urinary and reproductive organs)”
“4. Drugs affecting celluar function (drugs for tumors, radioactivity, cell activating drugs)”	“1. Drugs for the nervous system and sensory organs (central nervous system drugs etc.)” “2. Drugs for individual organ systems (drugs for cardiovascular, digestive organs, etc.)” “6. Drugs for pathogenic organisms (drugs for infectious diseases)”
“Deliberation”	–
“Priority review” “Sakigake designation” “Conditional early approval system”	“Expedited Review” “Notification of off-label use”
“Use of foreign data as evaluation material” “Implementation of International Joint Research”	“Application using only domestic data” “Clinical trial not conducted” “Use of foreign data as reference material”
“Foreign companies”	“Japanese Company”
“Orphan drugs”	“Public knowledge-based application”

*AIDS* Acquired immune deficiency syndrome, *PACS* Postmarketing all-case surveillance

### Reexamination results and how PACS results were reflected in safety measures

Of the 277 surveyed drugs for which PACS was required for their approval, 87 reported reexamination results. The reexamination results of the 87 drugs (31.4%) were all classified as Category 1, which means that there is no need to revise drug-approval conditions, indicating their usefulness, which is consistent with approval. In addition, after investigating whether safety measures were adopted after drugs became commercially available, we found that measures such as revising package inserts and providing information to medical institutions were adopted for 53 drugs. Of these, PACS results were reflected in the safety measures for 14 drugs (see Electronic Supplementary Table 1). The safety measures that were based on PACS results for these 14 drugs included content related to the warning label (1 drug), and most other content included minor changes, such as changes to the adverse events written in the package insert. The contents of the safety measures for the 14 drugs were as follows: warnings on the package insert (1 case); indication of possible adverse events on the package insert (8 cases); and others, including precautions for use and other precautions, careful administration, precautions for concomitant use (6 cases) in the package insert, and provision of information to medical institutions (4 cases).

## Discussion

### Key findings

To our knowledge, this manuscript is the first comprehensive survey analysis in English regarding drugs requiring PACS within 20-years in Japan. The results revealed that many drugs in the following categories, which are prevalent in a small number of patients at the time of new drug application, required PACS as an approval condition: anti-cancer drugs; those studied in international joint-research studies; those that include overseas clinical trial data in the application data package; and those that use priority examination systems, such as the Sakigake Designation system, conditional approval, or priority review. Moreover, PACS did not adversely affect the reexamination results, such as causing a drug’s approval to be revoked.

### Strengths and weaknesses

Implementation of postmarketing surveillance using PACS in Japan is not well known worldwide. Previous studies are limited, and recent research findings are nonexistent on this particular topic [13, 20]. Moreover, the scope and time frame of previous studies are limited, and no other long-term comprehensive researches, such as the present study, exist. Additionally, most related research results thus far have been published in Japanese [15]. In a previous comprehensive study, Mori et al. demonstrated that from 2000 to 2005, more drugs tended to require PACS as a condition for their approval [12]. However, the survey period was 6 years, and there was no indication of how the PACS results were reflected in the safety measures. In addition, with regard to how PACS results were reflected in safety measures, Suzuki et al. investigated whether the PACS results of anticancer drugs were reflected in their package inserts, and the results were found to be partially reflected, with most results being reflected in revisions to the package inserts due to serious adverse events [9]. However, this survey limits the target drugs to anticancer drugs, and there is no description related to the PACS results of other drugs that were approved in Japan.

The present study had limitation because this study was a retrospective survey of publicly available information and not a prospective study. And only drugs for which approval was obtained were surveyed, and drugs that had been discontinued or had not been approved were not included in the survey. Also, this study involves just a safety measures because of PACS nature, others, we don’t refer to the effectiveness.

### Interpretation

Herein, we review and compare the efforts of regulatory agencies regarding postmarketing safety measures in Japan. First, the conditions for approval after a drug becomes commercially available can generally be divided into three categories: (1) Mandatory additional clinical trials after approval (postmarketing clinical trials), (2) Limiting medical institutions or doctors who can use the drug for a certain period after approval (limitation of use), and (3) Collecting all information on patients who have used the drug for a certain period or until a certain number of patients is reached after approval (PACS). In other words, the approval conditions, including PACS, are part of the Risk Management Plan (RMP), which minimizes risks and conducts focused safety monitoring after approval of risk factors identified at the development/review stage. Since PACS is also an RMP component, the risk factors to which the approval conditions are attached are clearly indicated, and this is considered to determine the purpose and method of PACS. Hence, regarding this point, PACS use remains debatable.

PACS is considered to have commenced in 1999 [10]. In the present survey, the annual number of drugs for which PACS was conducted remained insignificant until 2005. However, in 2006, there were 10 drugs, and since then, the number has increased rapidly. One of the reasons for this is that since 2005, the problem of drug lag has become apparent and has emerged as a social issue, and regulatory agencies have begun to focus on measures to combat drug lag [21–23]. Many of the drugs covered by PACS are orphan drugs or those for serious diseases for which there are no existing effective drugs. Since it takes many years for a drug to reach the market, if sufficient validation studies are conducted, it is believed that the focus has shifted to postmarketing confirmation of efficacy and safety through comparative risk-benefit considerations based on regulatory science. It is also believed that this is partially attributable to the fact that the authorities did not order PACS when gefitinib-related problems with interstitial pneumonia were raised [24]. Incidentally, erlotinib (filed in 2006 and approved in 2007), which is a TKI like gefitinib, required PACS as a condition for drug approval. From the start, issues of risks and benefits should have been discussed; however, PACS might have been viewed as a scapegoat. On analyzing the percentage of all approved drugs for which PACS was a condition for their approval, in 2008 and in the preceding five years, over 20% of the approved drugs required PACS.

PACS collects comprehensive and unbiased clinical data at a relatively early stage, and there is great merit regarding risk minimization by providing feedback to medical professionals. Moreover, by requesting medical institutions to participate in PACS, a large number of reports with information such as that regarding adverse events can be obtained from those institutions [25]. However, when conducting PACS, there are no case-control comparisons, and methodological limitations, such as omission of data collection, are nonexistent [25]. In addition, safety measures, such as continuing case registration even after necessary cases have been collected, are confused with corporate research and investigation, thus complicating survey items. Consequently, the burden on resources of both the manufacturing and sales industries as well as of medical institutions, including preparations, emerges as a disadvantage [25]. Additionally, extraordinary costs is another problem of pharmaceutical companies. [25, 26].

### Further research

The Good Post-marketing Study Practice was amended in 2018 to adopt a comparative control group and the implementation of a database survey [27]. Database surveys are expected to be conducted as safety measures in the future. In addition, there has been an increasing number of cases in recent years where consent that is not legally required is obtained all the same [28]. First, PACS is likely to be valuable as a safety measure that can be adopted promptly without bias immediately after a drug enters the market, unlike database surveys. Especially for rare diseases, cancers, or rare and serious pediatric diseases, it is meaningful to spend time and financial resources on conducting PACS after a drug becomes commercially available. Similarly, in the early approval system (Sakigake Designation/conditional approval), data are often limited at the time of clinical trials. In this case, a verification of effectiveness may be necessary; it may be effective to conduct a survey that investigates the effectiveness and safety of the drugs examined in all cases. Unmet medical needs have been identified for more serious and rare diseases, and early approval is expected to be utilized to a greater extent in future drug development. Under such circumstances, PACS in Japan is considered to become even more meaningful. Methods that can obtain information regarding effectiveness in a way that is less burdensome on medical institutions, pharmaceutical companies, and even patients are warranted. This potentially includes methods such as conducting information research using a registry or limiting survey items by determining the necessary research questions for each disease.

## Conclusion

In this study, PACS did not adversely affect reexamination results, such as causing a drug’s approval to be revoked. Since there were some drugs for which the package insert was altered, or information was provided to medical institutions based on PACS results, PACS during the reexamination process was considered a meaningful postmarketing safety measure. However, when comparing factors such as the cost of implementing PACS and its quality with the response in the medical field resulting from the results of such surveillance, it is unclear whether PACS is a suitable safety measure from the perspective of cost effectiveness. For expedient and safe drug development in the future, more suitable methods will need to be considered, such as survey methods that utilize databases.

## Electronic supplementary material

Below is the link to the electronic supplementary material.

Supplementary file1 (DOCX 21 KB)
