# Supplementary material for: Postmarketing all-case surveillance trends and contribution to safety measures of drugs approved in Japan: a cross-sectional survey in 1999–2019
Source: Int J Clin Pharm. 2022 Nov 2;45(1):108–16. doi: 10.1007/s11096-022-01461-0 (PMC9938803; doi:10.1007/s11096-022-01461-0)
Supplement: Supplementary file 1 — Supplementary file1 (DOCX 21 KB) [file 11096_2022_1461_MOESM1_ESM.docx]

**Supplementary Information**

**Postmarketing all-case surveillance trends and contribution to safety measures of drugs approved in Japan: a cross-sectional survey in 1999–2019**

*International Journal of Clinical Pharmacy*

Minami Nakao^1^, Yuri Nakamura^1^, Masafumi Shimokawa^2^, Hideki Maeda^1^*

^1^Department of Regulatory Science, Faculty of Pharmacy, Meiji Pharmaceutical University, Tokyo, Japan

^2^Faculty of Pharmaceutical Sciences, Sanyo-Onoda City University, Yamaguchi, Japan

***Corresponding author**: Hideki Maeda

Email: maeda@my-pharm.ac.jp

**Supplementary Table S1** List of safety measures reflected by postmarketing all-case surveillance

| **Product No.** | **Generic Name** | **Brand Name(s)** | **Indication(s)** | **Year Approved** | **Reexamination Period (Years)** | **Section Name** | **Details** |
| --- | --- | --- | --- | --- | --- | --- | --- |
| 1 | Piracetam | Piracetam UCB Myocalm Solution Myocalm Solution Taiho | Combination therapy with antiepileptics, etc., for cortical myoclonus | 1999 | 10 | Summary of Drug Use Results Surveillance_Safety | Judged that Alerts concerning hepatic impairment, ALP elevation, and CK elevation, which affected a large number of the cases gathered, are required in the Package Insert. |
|  |  |  |  |  |  | Summary of Drug Use Results Surveillance_Efficacy | Applicant's response was inadequate … Issue another Alert to healthcare providers in future |
|  |  |  |  |  |  | Summary of Drug Use Results Surveillance_Special Populations | Concerning children, revise the Precautions for Use. Concerning older people, Alert already issued for patients with renal impairment or liver impairment |
| 2 | Human Activated Protein C, Freeze-Dried Concentrated | Anact C for Injection 2,500 Units | The following conditions caused by congenital protein C deficiency | 2000 | 10 | Summary of Drug Use Results Surveillance_Priority Surveillance Items | Described in both the Interactions (Precautions for Co-Administration), Precautions for Use section, and the Important Basic Precautions sections |
| 3 | Etanercept (Genetical Recombination) | Enbrel for Subcutaneous Injection 25 mg | Rheumatoid arthritis (only in the case of inadequate response to existing therapies) | 2005 | 8 | Drug Use Results Surveillance_Safety | Concerning patients in specific conditions (Translator's note: is this the correct understanding?), descriptions included in the Precautions for Use section of the Package Insert, and Alert issued. |
|  |  |  |  |  |  | Drug Use Results Surveillance_Priority Survey Items | Malignant neoplasms described in the Warnings and Important Basic Precautions sections of the Package Inset, and Alert issued.  Described in the Clinically Significant Adverse Drug Reactions section |
|  |  |  |  |  |  | Drug Use Results Surveillance_Special Populations | Already described in the Warnings and Precautions for Use sections of the Package Insert; no particular issues requiring a response are considered to be apparent |
| 4 | Busulfan | Busulfex for Intravenous Infusion 60 mg | (Underlined part added to this version) 1．Pretreatment prior to allogeneic hematopoietic stem-cell transplantation 2．Pretreatment prior to autologous hematopoietic stem-cell ransplantation for Ewing's sarcoma family tumor or neuroblastoma | 2006 | 10 | Safety_Drug Use Results Surveillance_Priority Surveillance Items | Also published on the website of the Japan Society for Hematopoietic Stem Cell Transplantation Described in the Adverse Drug Reactions (Overseas Clinical Trials) section of the Package Insert Described in the Important Basic Precautions and Clinically Significant Adverse Drug Reactions sections of the Package Insert, and Alert issued |
| 5 | Tacrolimus Hydrate | PROGRAF Capsules 0.5 mg PROGRAF Capsules 1 mg | Lupus nephritis (in the case of inadequate response to steroid administration or if this is prevented by adverse drug reaction) | 2007 | 10 | Safety_Specified Drug Use Results Surveillance_Priority Surveillance Items | Considered appropriate to delete the statement under "Other Precuations" in the Precautions for Use section of this drug's Package Insert that "There are few results of clinical trials lasting more than 8 weeks, and the safety of long-term administration has not been established." |
| 6 | Idursulfase (Genetical Recombination) | ELAPRASE for Intravenous Infusion 6 mg | Type II mucopolysaccharidosis | 2007 | 10 | Safety_Specified Drug Use Results Surveillance_Priority Surveillance Items | Considered to be necessary to continue the Alert concerning regular anibody testing in the Package Insert in future. |
| 7 | Nitric Oxide | INOflo for Inhalation 800 ppm | Improvement of neonatal hypoxic respiratory failure associated with pulmonary hypertension | 2008 | 10 | Safety_Specified Drug Use Results Surveillance | Taking account of the nature of adverse drug reactions, Package Insert revised, and safety information distributed to healthcare providers as informational material. |
| 8 | Erlotinib Hydrochloride | Tarceva Tablets 25 mg Tarceva Tablets 100 mg | ○Nonresectable recurrent or advanced non-small-cell lung cancer that is aggravated following chemotherapy  ○Nonresectable pancreatic cancer (Underlined part added) | 2011 | Remaining period (from July 1, 2011 to October 18, 2015) | Safety_Specified Drug Use Results Surveillance [Non-Small-Cell Lung All-Case Surveillance (ML21590)] | Alert to be issued in the Other Precautions of the Precautions for Use section of the Package Insert. |
|  |  |  |  |  |  | Safety_Specified Drug Use Results Surveillance (Pancreatic Cancer All-Case Surveillance [TAR1101]) | Alert issued in the Other Precautions of the Precautions for Use section of the Package Insert. |
| 9 | Bosentan Hydrate | Tracleer Tablets 62.5 mg | Pulmonary arterial hypertension (WHO functional classification Class II, III, or IV) | 2012 | 10 | Summary of Specified Drug Use Results Surveillance_Safety_Demographic Factors Affecting Safety | Alerts in the Warnings and the Precautions for Use Concerning Dosage and Administration sections concerning regular liver function testing and measures to take in the event of abnormal liver function test results: at this point, a new Alert is considered unnecessary; however, distribution of informational materials for healthcare providers, including the dose reduction/withdrawal criteria for this drug, to continue. |
| 10 | Monteplase (Genetical Recombination) | Cleactor Injection 400,000 Cleactor Injection 800,000 Cleactor Injection 1,600,000 | Lysis of coronary artery thrombus in acute myocardial infarction (within 6 h post-onset) Lysis of pulmonary artery thrombus caused by acute pulmonary embolism in the presence of hemodynamic instability (Underlined part added or changed) | 2005 | 10 | Summary of Specified Drug Use Results Surveillance_Special Populations | Cautious Administration section: changed |
|  |  |  |  |  |  | Summary of Specified Drug Use Results Surveillance_Priority Surveillance Items | Precautions for Co-Administration section: Described with Alert |
| 11 | Zinc Oxide Hydrate | NOBELZIN Capsules 25 mg NOBELZIN Capsules 50 mg | Wilson's disease (hepatolenticular degeneration) | 2008 | 10 | Safety_Specified Drug Use Results Surveillance_Appearance of Adverse Drug Reactions | Added to the Package Insert during the reexamination period and Alert issued. Letter providing information on correct use distributed, and Alert issued (February 2017) |
| 12 | Vancomycin Hydrochloride | Vancomycin Ophthalmic Ointment 1% | **Indicated Bacterial Species** Vancomycin-sensitive methicillin-resistant *Staphyloccocus aureus* (MRSA), methicillin-resistant *S. epidermis* (MRSE)  **Indications** The following diseases with an inadequate response to existing therapies: conjunctivitis, blepharitis, tarsadenitis, dacryocystitis | 2009 | 10 | Safety _Drug Use Results Surveillance | October 2010: Corneal damage added to Clinically Significant Adverse Drug Reactions section |
| 13 | Infliximab (Genetical Recombination) | Remicade for Intravenous Infusion 100 | The following diseases with an inadequate response to existing therapies: 　Rheumatoid arthritis (including the prevention of structural joint damage) 　Refractory uveoretinitis associated with Behçet's disease **Ankylosing spondylitis** Treatment or maintenance therapy for Crohn's disease exhibiting any of the following conditions (only in the case of inadequate response to existing therapies): 　Moderate to severe disease activity 　External fistula (Underlined part added) | 2010 | 10 | Safety_Specified Drug Use Results Surveillance | Tuberculosis described in Clinically Significant Adverse Drug Reactions section of Package Insert, and caution continued |
| 14 | Dasatinib Hydrate | Sprycel Tablets 20 mg Sprycel Tablets 50 mg | 1. Chronic myelogenous leukemia 2. Recurrent or refractory Philadelphia-chromosome-positive acute lymphoblastic leukemia (Underlined part added) | 2011 | 10 | Safety_Drug Use Results Surveillance | Shingles present, but already described in the Precautions for Use section of the Package Insert |
